# Remote Sensing and Wetland Ecology: a South African Case Study

**DOI:** 10.3390/s8053542

**Published:** 2008-05-26

**Authors:** Els R. De Roeck, Niko E.C. Verhoest, Mtemi H. Miya, Hans Lievens, Okke Batelaan, Abraham Thomas, Luc Brendonck

**Affiliations:** 1 Laboratory of Aquatic Ecology and Evolutionary Biology, Katholieke Universiteit Leuven, Charles Deberiotstraat 32, 3000 Leuven, Belgium; 2 Laboratory of Hydrology and Water Management, Ghent University, Coupure links 653, 9000 Ghent, Belgium; E-mail: niko.verhoest@ugent.be; 3 Department of Hydrology and Hydraulic Engineering, Vrije Universiteit Brussel, Pleinlaan 2, 1050 Brussels, Belgium; 4 Department of Earth and Environmental Sciences, Katholieke Universiteit Leuven, Celestijnenlaan 200e - bus 2410, 3001 Heverlee, Belgium; 5 Department of Earth Sciences, University of the Western Cape, P Bag X17, Bellville 7535, Cape Town, South Africa

**Keywords:** Wetland monitoring, wetland distribution and density, wetland ecology, Landsat, Envisat

## Abstract

Remote sensing offers a cost efficient means for identifying and monitoring wetlands over a large area and at different moments in time. In this study, we aim at providing ecologically relevant information on characteristics of temporary and permanent isolated open water wetlands, obtained by standard techniques and relatively cheap imagery. The number, surface area, nearest distance, and dynamics of isolated temporary and permanent wetlands were determined for the Western Cape, South Africa. Open water bodies (wetlands) were mapped from seven Landsat images (acquired during 1987 – 2002) using supervised maximum likelihood classification. The number of wetlands fluctuated over time. Most wetlands were detected in the winter of 2000 and 2002, probably related to road constructions. Imagery acquired in summer contained fewer wetlands than in winter. Most wetlands identified from Landsat images were smaller than one hectare. The average distance to the nearest wetland was larger in summer. In comparison to temporary wetlands, fewer, but larger permanent wetlands were detected. In addition, classification of non-vegetated wetlands on an Envisat ASAR radar image (acquired in June 2005) was evaluated. The number of detected small wetlands was lower for radar imagery than optical imagery (acquired in June 2002), probably because of deterioration of the spatial information content due the extensive pre-processing requirements of the radar image. Both optical and radar classifications allow to assess wetland characteristics that potentially influence plant and animal metacommunity structure. Envisat imagery, however, was less suitable than Landsat imagery for the extraction of detailed ecological information, as only large wetlands can be detected. This study has indicated that ecologically relevant data can be generated for the larger wetlands through relatively cheap imagery and standard techniques, despite the relatively low resolution of Landsat and Envisat imagery. For the characterisation of very small wetlands, high spatial resolution optical or radar images are needed. This study exemplifies the benefits of integrating remote sensing and ecology and hence stimulates interdisciplinary research of isolated wetlands.

## 1. Introduction

Remote sensing and GIS techniques are both increasingly valued as useful tools for providing large-scale basic information on landscape characteristics [1]. They are used for habitat and species mapping, biodiversity determination, land change detection, monitoring of conservation areas, and the development of GIS layers [2-8]. In many cases, remote sensing data can partially replace the often time consuming and expensive ground surveys [2, 9]. Also change detection of the earth's surface can be investigated due to the availability of long-term data [10-12].

Remote sensing offers a cost efficient means for delineating wetlands over a large area at different points in time and can provide useful information on wetland characteristics [5, 9, 13, 14]. Based on various remote sensing data types, many methods for delineating water bodies have been described [5, 15]. Wetland delineation involves most often the use of aerial photographs and airborne or satellite remotely sensed data [5, 15]. In the past, visual interpretation of wetlands from maps, aerial photography, and hard copy of satellite images have been used extensively [5, 16]. Currently, also digital image processing is used [2]. There is no standard method for computer-based wetland classification [5, 17]. Landsat, SPOT, AVHRR, IRS, and radar systems are the most frequently used satellite sensors for wetland detection [5].

On optical imagery, clear open water bodies are relatively easy to detect by means of computer aided classification, since water has a characteristic spectral reflectance. The most distinctive feature is the energy absorption at near-IR wavelengths and beyond [16]. Characteristics like water quality, turbidity and chlorophyll contents can also be determined using optical remote sensing techniques, but are more complicated to assess [16, 18, 19].

Unlike optical systems, radar is an active sensing device. It transmits short bursts of electromagnetic (EM) radiation to a surface target and measures the energy response returned from that target [16]. The response of the signal largely depends on the roughness of the illuminated area. A very smooth surface, like an open water body, reflects the signal away from the radar, resulting in a very weak response [20]. Contrarily, on very rough surfaces, such as vegetated soils, incident EM signals interfere and are ‘scattered’ in all directions, including the direction of the radar antenna [20]. This physical behaviour implies that a very simple and straightforward distinction between smooth open water surfaces and rough dryland surfaces can be established by means of threshold criteria. Another promising aspect of the active radar sensor is its independency of solar illumination [20]. As such, images can be acquired day and night. Moreover, the microwave signal, with a frequency ranging between 220 MHz and 40 GHz, is not absorbed by clouds or haze, as optical signals are.

Despite the potential of wetland delineation by remote sensing, wetlands are in general not well characterised, especially considering their ecological importance and vulnerability. Worldwide, there is a need for continued wetland inventories as small water bodies have often been underemphasized, and many inventories are therefore unreliable [15, 22]. The use of remote sensing in fundamental temporary and permanent wetland ecology, moreover, is currently not widespread, but has a large potential. Some biologists claim that spatial scales of remote sensing and scales usually covered by ecological or evolutionary research do not match, thus creating a perception problem [8], limiting the use of remote sensing techniques in biological studies.

The aim of this study is to indicate that elementary and relatively cheap imagery and basic remote sensing techniques can substantially improve the knowledge on characteristics of temporary and permanent wetlands. In this study isolated open water wetlands in the Cape region of the Western Cape were characterised from seven Landsat images using supervised classification methods. Classification results of the Landsat imagery were compared with those of an Envisat image. Ecologically relevant traits (surface area, distance, dynamics, total number, and fraction of temporary and permanent wetlands) were investigated and discussed within the scope of wetland ecology. The effectiveness as well as the limitations of this straightforward remote sensing study, as an addition to ecological research, were evaluated.

## 2. Materials and Methods

### 2.1 Optical wetland detection

Isolated open water wetlands were classified from seven Landsat TM and ETM+ images acquired on 9 January 1987 (summer), 16 October 1990 (winter), 3 June 1999 (winter), 4 December 1999 (summer), 31 July 2000 (winter), 24 February 2001 (summer), and 3 June 2002 (winter). Images were downloaded from the Global Land Cover Facility (GLCF) website or purchased from the United States Geological Survey Organization (USGS). The study area is located in the Cape Region of the Western Cape Province, South Africa (within latitude 33° 03′ to 33° 52′ South and longitude 17° 57′ to 19° 05′ East). It has a Mediterranean climate, receives much of its rainfall in winter months, and has relatively dry summers [23].

Ground truth data were collected for the larger (from 0.32 hectare onwards) wetlands in the area by field surveys in 2004 and 2005 and supplemented with information from topographical maps obtained from the South African Chief Directorate of Surveys and Mapping. Most of the vegetation of these larger water bodies was situated at the edges (personal observation). Band 4, which showed a strong contrast between water bodies and other land features, was used to define at least 25 training sites for each land cover type (fresh water, sea, mountains, two types of vegetation, city, and sand dunes). The training data were randomly selected from this database, while the non-selected data points (50% of the database) were used as validation data for the accuracy analysis. Bands and band ratios with best optical view of water bodies were visually selected, enhanced, and used to create signature files. Three supervised classification methods (maximum likelihood, minimum distance, and Fisher) were compared. Unwanted land features with similar or near similar reflectance as freshwater wetlands (such as rivers, sea, and shadows) were masked out of each image, ensuring reliable comparison of all images. The position of shadow in each image was determined by means of a Digital Elevation Model (DEM), elevation level of the sun, and sun azimuth at the time of image acquisition.

The user's, producer's, overall accuracy, and Kappa Index of Agreement (KIA) for wetland classification were computed on the validation data. Classification accuracy assessments determine the degree of correspondence between the classified pixels and the reality (ground truth) (for more information on each of these measures, see [16]). The classification method with highest accuracy and best visual results was used for all analyses. Delineated wetlands smaller than two pixels were not taken into account in further analyses, as chance for inaccurate classification was high due to interference with other land features. The smallest detectable wetlands thus had a surface area of about 0.16 hectare. All classifications and accuracy analyses were performed in IDRISI Andes [24].

The number of classified isolated open water wetlands and their corresponding surface area were determined by standard techniques. Any relation was analysed between the number of detected wetlands and the cumulative rainfall respectively over three, six and twelve months before image acquisition (Spearman Rank correlation, Statistica 7 [25]). The average Euclidean distance from each wetland to the nearest wetland as well as the distance between each pixel and the nearest wetland were obtained. The number and surface area of temporary and permanent wetlands in 2000-2001 were determined through a series of image manipulations. To reveal the temporary wetlands in the area, the summer image, normally containing only permanent wetlands, was subtracted from the winter image, containing temporary and permanent wetlands. The periphery of permanent wetlands is often dry in summer. To prevent misclassification of these periphery-pixels as individual temporary wetlands, a buffer of 85 m (about three pixels) surrounding each permanent wetland was masked out of the temporary wetland delineation. A field survey in the winter of 2005 provided an estimate of the number of wetlands in the study area that were smaller and larger than the resolution of Landsat TM (about 0.081 hectare). These analyses were conducted in IDRISI Andes [24] and ArcGIS 9.2 [26].

### 2.2 Radar wetland detection

The radar image used is a C-band (5.3 GHz) Envisat Advanced Synthetic Aperture Radar (ASAR) HH-polarised image, acquired on June 26 in 2005. The swath operation mode employed during acquisition was I2, characterised by an incidence angle ranging between 19.2° and 26.7°. The spatial resolution of the ASAR image is 30 m both in azimuth and range direction. However, the pixel spacing is 12.5 m by 12.5 m.

In a first pre-processing stage, the raw ASAR image was calibrated following the method described by ESA [27]. After calibration, the image was co-registered with the Landsat ETM+ image of June 2002, using a simple two-dimensional linear regression polynomial. The RMSE for the georeferencing was 3.8 pixels, corresponding with 47.5 m on ground. Furthermore, to reduce the speckle noise, which is inherent to radar data, the Enhanced Frost filter with a window size of 5 by 5 pixels was applied [28-29]. A last pre-processing step was a two-level bottom-up region merging segmentation, performed in eCognition 3.0 [30]. This algorithm requires three input parameters: scale, colour/shape weights, and smoothness/compactness weights. The scale parameter is indirectly related to the outcome segment size. In this study, the segmentation was performed on two hierarchic scale levels. A first coarse level was derived with a large scale parameter of 40. In this level, segments were able to fit very large objects in entirety. Yet, most small objects like many temporary wetlands were merged with dryland objects in single segments. Within this first level, a second segmentation level was created on the basis of a finer scale, equal to 25. In this refined level, small wetlands were still preserved. Both levels will later be used for the statement of threshold criteria for classification. The other parameters were optimised during a trial and error study on simulated radar imagery. For this study, the optimal colour/shape weighting, which addresses the relative influences of spectral information versus outcome segment shapes, is equal to 0.9/0.1. This results in segments with a narrow spectral value, which may be of irregular shape. The smoothness/compactness contributes to the jaggedness of the outcome segments and was also set as 0.9/0.1, allowing segments to comprise the natural outlines of features.

Image segments were classified as wetland and dryland by means of simple threshold criteria on segment value and segment shape-index, defined by the perimeter over area. The shape-index threshold aims at excluding large dryland objects with similar backscatter values as open water and highly irregular shapes from the wetland classification, such as airports, sand dune areas and rivers. In the small scale level, such large objects are often covered by several adjacent segments with a reduced shape-index instead of one large jagged segment with a high shape-index. Therefore, the shape threshold is established on the large scale level. On the other hand, the threshold on segment value is applied on the small scale level, as in this level most small wetlands are formed by individual segments, through which their spectral information content is preserved. Both thresholds were optimised on simulated imagery by iteratively increasing the threshold values and computing the KIA. The optimal threshold on segment value equals -15.38 dB, whereas the most adequate shape-index threshold equals 2.2. This shape-index agrees with the natural shape of most wetlands in the Western Cape, being of circular or ellipse shape, and thus characterised by a small index. However, linear wetland features that are probably caused by the construction of roads may disappear using this technique. Yet, the removal of sand dunes and airports from the classification output was thought to be of more importance than the preservation of some linear wetland features along roads.

### 2.3 Comparison of optical with radar classification

The classification result on the Envisat ASAR image of June 2005 is compared by means of a cross-classification table with the result on the Landsat image of June 2002, as this Landsat image of winter 2002 is the most recent one. Because of steep relief in the South of the study area, which severely hampers radar image interpretation, the comparison will only be performed on a mainly flat sub-area in the North. There was a relatively small difference in the amount of rainfall in the months preceding the image acquisition in 2002 (Landsat) and 2005 (Envisat). The cumulative rainfall three and six months before image acquisition in 2002 was respectively 127 mm and 207 mm; for 2005 this was respectively 196 mm and 223 mm. According to the landowners in the area, no large changes in the positioning of the wetlands occurred from 2002 to 2005.

## 3. Results

### 3.1 Classification on optical imagery

The maximum likelihood classification method had the highest mean overall accuracy and user's accuracy for wetland detection, although the difference with the Fisher and minimum distance methods was small (Table 1). Fisher classification method had a slightly higher producer's accuracy and categorical KIA than the maximum likelihood classification method (Table 1). The accuracy for wetland detection by the maximum likelihood method was high for all seven Landsat images, with a user's, producer's and overall accuracy, and categorical Kappa Index of Agreement for wetland detection higher than 0.91.

**Figure 1.**
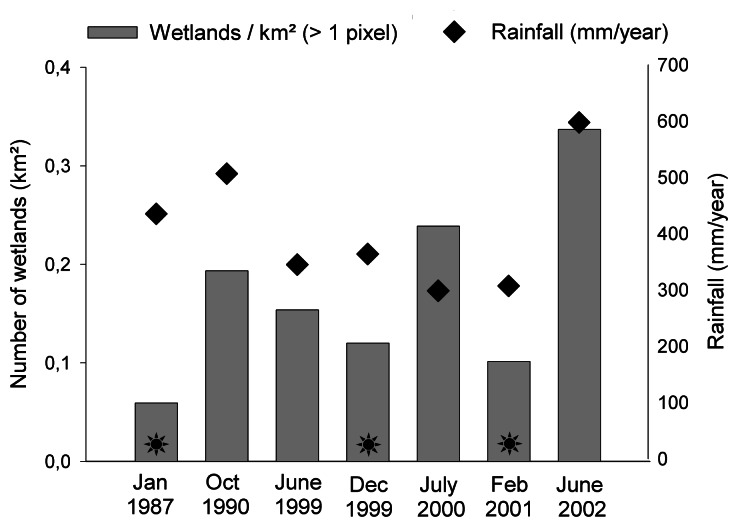
Number of delineated wetlands per km^2^. Sun symbols indicate images taken during summer. Diamonds indicate the cumulative amount of rainfall over twelve months before the date of image acquisition (mm/year).

Images taken in summer revealed on average less wetlands (0.09 per km^2^) than those taken in winter (0.23 per km^2^; Figure 1). The number of winter wetlands fluctuated in time. For the wetlands detected in the summer images, no correlations were detected with cumulative rainfall respectively over three, six and twelve months before image acquisition (Spearman Rank correlation: p > 0.05). Similar results account for the winter images. Most wetlands were detected in winter 2002, when rainfall was relatively high (Figure 1). Visual interpretation of classified images revealed the positioning of many wetlands along roads in the winter images of 2000 and 2002, and to a lesser extent in 1999 (respectively about 9%, 7% and 4% of the detected wetlands).

**Figure 2.**
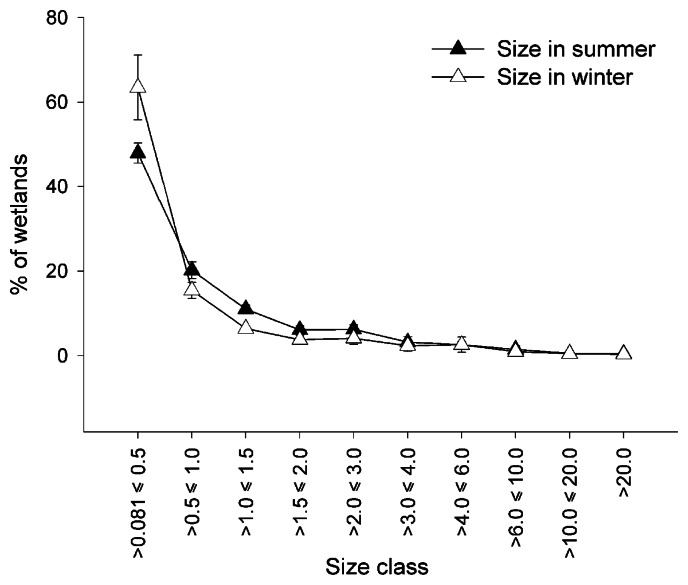
Fraction of detected wetlands belonging to different size classes (in ha) in summer and in winter, with indication of the standard deviation.

In general many small and only few large wetlands were detected in both summer and winter images (Figure 2). On average 68% of summer wetlands and 79% of winter wetlands were smaller than one hectare. More small wetlands were detected in winter than in summer (Figure 2). The ground survey in winter 2005 revealed that about 73% of the wetlands (which were mostly temporary) in the study area could not be detected, since they were smaller than the resolution of the Landsat imagery (0.081 ha). Another 15% of wetlands observed in the field covered an area of about one pixel. Due to a high chance of misclassification they were excluded from this study. Consequently, only about 12% of wetlands present in the study area could be detected by Landsat classifications.

**Figure 3.**
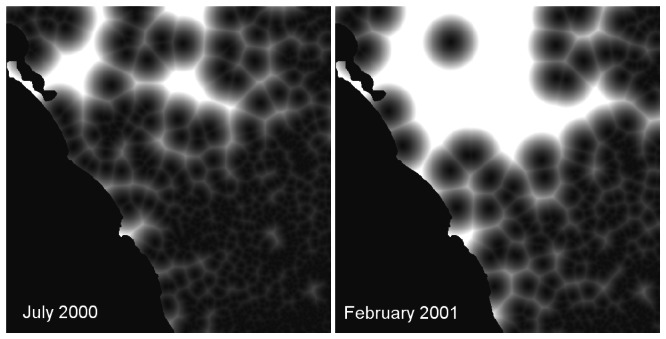
Visualisation of the distance of each pixel to the nearest wetland in the winter of 2000 (left) and in the summer of 2001 (right); light shading = long distance, dark shading = short distance to the nearest wetland.

The average distance between each wetland and the nearest neighbouring wetland was 763 m in summer (standard deviation: 126 m) and 441 m in winter (standard deviation: 114 m). The mean Euclidean distance from each pixel to the nearest wetland was larger in summer images (average 4305 m) than in winter images (average 1748 m), which is visualised in Figure 3 for the winter of 2000 and summer of 2001. Wetlands were not evenly distributed over the study region (Figure 3).

In 2000-2001, on average 0.17 temporary wetlands per km^2^ were delineated, while less permanent wetlands were found (0.09 per km^2^). Temporary wetlands were smaller (average 0.39 ha) than permanent ones (average 1.29 ha; size in winter).

### 3.2 Comparison of optical with radar classification

**Figure 4.**
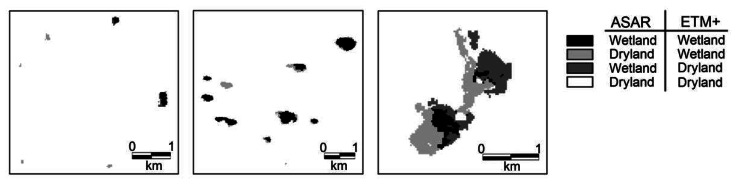
Details from the cross-classification of the maximum likelihood Landsat ETM+ classification of June 2002 and the Envisat ASAR classification of June 2005. The left part shows a region in the East of the study area containing few small to medium-sized wetlands; the center inset illustrates classification results over a vast area with medium-sized to large wetlands, located in the center of the study area; the right part covers a very large salt pan near the Atlantic coast.

**Figure 5.**
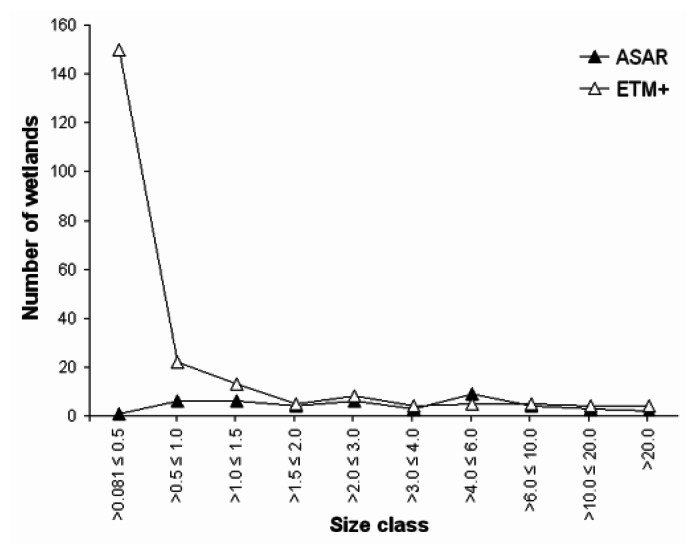
Number of detected wetlands in the study area used for comparison (see materials and methods), as a function of wetland size class (ha) for the Envisat ASAR image of June 2005 and the Landsat ETM+ image of June 2002.

The classification result on the Envisat ASAR image of June 2005 is compared with the result of the Landsat ETM+ image of June 2002 by means of a cross-classification table (Table 2) and cross-classification image, of which three informative insets are illustrated in Figure 4. Figure 5 shows a frequency distribution of detected wetlands in function of wetland size class (ha) for both the Landsat and Envisat classification.

As indicated in Table 2, more wetland pixels are detected on the Landsat image (4336) than on the Envisat image (2899). The frequency distribution (Figure 5) clearly shows that almost no wetlands smaller than 0.5 ha can be detected on the Envisat imagery. For wetland classes from 1.5 ha onwards, the results on ETM+ and ASAR HH imagery are very similar.

## 4. Discussion

This study revealed that basic remote sensing techniques can be used as a tool to acquire ecologically relevant information on temporary and permanent open water wetlands that are larger than the resolution of the classified images.

In the optical study, the maximum likelihood classification procedure had the highest overall and user's accuracy for detection of open water wetlands, while the Fisher classification method had a slightly higher producer's accuracy and categorical KIA. Since the maximum likelihood procedure is one of the most commonly used supervised classification methods for wetlands [5] and since its accuracy is comparable to that of other procedures, it was used for all further analyses. The high accuracy of the classifications indicates that basic remote sensing techniques are sufficient to detect open water wetlands on Landsat imagery, due to the distinctive spectral characteristics of open water.

Logically, more wetlands were classified in the rainy season (winter images; on average 0.23 per km^2^) than in the dry season (summer images; on average 0.09 per km^2^). On average, 68% of the detected summer wetlands and 79% of the winter wetlands were smaller than one ha. The distance to the nearest detectable wetland was larger in summer (on average 763 m) than in winter (on average 441 m). These seasonal differences most probably result from the many temporary wetlands that are flooded in the area during winter. In comparison to permanent wetlands, more, but on average smaller, temporary wetlands were detected in 2000-2001. However, some permanent wetlands that were too small to detect during summer could probably become larger and detectable in winter. The occurrence of these wetlands may therefore be misinterpreted and falsely contribute to this apparent higher number of temporary wetlands. To evaluate these deviations, high spatial resolution satellite imagery or aerial photographs are indispensable.

The number of wetlands fluctuated over time (1987 – 2002). There was however no significant relation between rainfall pattern and the amount of wetlands detected in the winter or summer images. Probably the differences in rainfall pattern are not large enough to significantly influence the occurrence of the larger, detectable wetlands. On the other hand, for the very small temporary wetlands that were undetectable by Landsat, relatively large differences in their amount and size occur between years (personal observation in the field), probably related to precipitation patterns. In the winter of 2000 and 2002, a higher number of wetlands was detected. The construction of roads on embankments probably created these additional wetlands, since visual interpretation of the classified images revealed their linear positioning alongside roads. Other factors, such as temperature and groundwater fluctuations, could also have influenced the amount of wetlands over the investigated years.

Classification on the Envisat image by means of threshold criteria on segment value and segment shape-index also appeared to be a simple and straightforward method for the delineation of open water bodies in the Western Cape. However, results illustrated that considerably less small wetlands were detected in Envisat imagery compared to Landsat imagery. The major cause for the underestimation of wetland occurrence in the radar scene is the application of spatially averaging noise suppression techniques as filtering and segmentation. This can also be revealed from the left part of Figure 4, where the two larger wetlands at the top right are detected on both images, whereas all smaller wetlands at the bottom left are only discerned on ETM+ imagery. Larger wetlands are thus accurately detected on both optical and radar images, while small wetlands are not. As a consequence, small wetlands, which are often temporarily inundated, are no longer distinguishable and calculations of ecological parameters must hence be interpreted with care. Cross-classification results (Table 2) reveal that a higher number of wetland pixels are detected with Landsat (4336) than with Envisat (2899), probably due to the loss of many small wetlands during Envisat image processing.

Only a relatively small amount of wetland pixels were detected by both radar and optical image classifications (Table 2). This low number is probably caused by severe displacements. Since a radar device measures distances from ground targets to the radar antenna, variations in topography can cause features to be shifted. Features lying at higher (lower) elevation will be located more (less) near-range on the image, which is not corrected for during georeferencing. Moreover, optical images may present a complete reverse shift. An increase in the elevation of a feature causes its position on the image to be displaced radially outward from the principal point, a shift referred to as ‘relief displacement’ [16]. A specific example of such displacement is shown in the right part of Figure 4, covering a large salt pan near the Atlantic coast. For this wetland, containing 923 pixels (i.e. the average area of the wetland as sensed with Landsat and Envisat), only 244 pixels were detected in common. Due to these displacements, the direct use of classifications of both optical and radar imagery in one study must be cautiously interpreted.

In addition, also the comparison between the classifications of Landsat and Envisat images has to be interpreted with care due to the time lag between the image acquisitions (2002-2005). In this time span several wetlands may have been created or lost. According to landowners, however, there were no large changes in land use in that period. Possibly fewer wetlands were water logged on the 2002 Landsat image compared to the 2005 Envisat image, since there was less precipitation in the first six months preceding the 2002 image acquisition.

Monitoring of wetland characteristics by remote sensing can provide important information for ecological research and for the development of conservation measures. Little is currently known on the number, surface area, and distribution patterns of temporary and permanent wetlands in the Western Cape, despite the importance of such knowledge for wetland ecology and management. In general, classification of Southern African inland waters was restricted due to the high cost [11]. In accordance with our results, Silberbauer and King [31] have determined many small wetlands in the same study area. These authors detected about 0.17 temporary and permanent wetlands per km^2^ (deducted from maps), which is slightly less than detected in our optical study (about 0.23 wetlands per km^2^). Comparing both studies, however, should be done with care, since the work of Silberbauer and King [31] was based on 1:50 000 topographical survey maps from 1953 to 1987. At present, the National Land Cover project is mapping wetlands and other land features in South Africa (using Landsat 7 ETM+ imagery), creating additional valuable information.

The number of wetlands detected by Landsat or Envisat classification can be important for the study of the ecology and for the conservation of local and migrating wading birds, which currently occur in large numbers in the studied area [32] and especially feed in the larger water bodies (personal observation). Reduction of numbers of wetlands, for example due to water abstraction or climate change, may negatively affect several of these species, such as flamingos. The number, surface area, isolation, and density of wetlands can also influence the metapopulation and metacommunity structure and the persistence of amphibians and invertebrates [33-34]. Large branchiopods, which are typical inhabitants and flag ship species of temporary waters, for example, can be genetically isolated even over short distances [35], illustrating the importance of distance between wetlands for invertebrate populations. Vanschoenwinkel and co-workers [36] furthermore measured that also at (invertebrate) community level, dissimilarity increased with distance among rock pools. Semlitsch and Bodie [37] observed that a loss of small wetlands in the Atlantic Coastal Plain (U.S.A.) would increase the distance to the nearest wetland, impeding the rescue effects of amphibians at the metapopulation level.

A ground survey in winter 2005 revealed that at least 88% of wetlands present in the area were not detected by our remote sensing study, due to their small size. Even more wetlands are missed out in the Envisat classification. Most of these undetectable wetlands were of a temporary nature (personal observation). All analyses and conclusions in this study thus apply only to the larger detectable isolated open water wetlands. Research on the effect of wetland isolation on amphibian communities requires very high spatial resolution images, since small wetlands are important habitats for amphibians [37] and may act as stepping-stones between the larger ones [38-39]. In this respect, high spatial resolution images, such as IKONOS images for optical studies and RADARSAT-I and future RADARSAT-II imagery for radar purposes, would give spatially more detailed results [14], if we assume that no further complications arise with the use of these images. However, these images have a small footprint and there are no long time series available yet. Moreover, they are at present expensive and many images are needed for the detection of small temporary wetlands due to their often short inundation period. The resolution of Landsat and Envisat is sufficient, on the other hand, for research on habitats important for several water bird species that do not occur, or only in very low abundances, in small wetlands [40]. Satellite imagery can be especially useful for many studies on habitats of highly mobile organisms such as water birds, as they require data over a large tract of land [41]. The resolution of the images used to delineate wetlands hence should be adapted to the size of the studied wetlands.

Despite the usefulness of remote sensing data, the basic knowledge of many ecologists on remote sensing and GIS is still poor, creating the need for more extensive training. In addition, specialists are needed in order to detect more detailed information such as wetland inundation period or different types of vegetated wetlands from optical or radar images. In general, it is important that ecologists are aware of the possibilities and limitations of remote sensing techniques. Depending on the study systems and the resolution of the images used for classification, valuable information on wetland ecosystems can be gathered through cooperation between remote sensing specialists and ecologists.

## 5. Conclusions

Basic descriptive data of open water temporary and permanent wetlands can be generated through relatively cheap remote sensing imagery and standard GIS techniques, which can be important for ecological research and hence for the creation of conservation measures. However, since many of the temporary wetlands are very small in the Western Cape, the resolution of Landsat or Envisat images is insufficient to be used as an exhaustive monitoring tool from space. Small wetlands (smaller than 1.5 ha) were even more difficult to discern with Envisat ASAR imagery than with Landsat. Very high accuracy images (like IKONOS or Radarsat I/II) would probably yield more detailed results, but the high cost and the lack of long term time series are at present restricting factors for their use by biologists. To fully understand wetland ecosystem functioning in a broader context, interdisciplinary research is of high priority. We therefore strongly encourage the integration of remote sensing and aquatic ecology, and the expansion of remote sensing knowledge to more ecologists.

## Figures and Tables

**Table 1.** Accuracy analysis of wetlands classified by maximum likelihood (MaxLike), minimum distance (MinDist), and Fisher classification methods. Mean values for all classified images are presented.

	Maxlike	MinDist	Fisher
User's accuracy	0.943	0.912	0.942
Producer's accuracy	0.970	0.969	0.983
Overall accuracy	0.999	0.998	0.998
Categorical KIA	0.970	0.967	0.986

**Table 2.** Cross-classification table of the maximum likelihood Landsat classification of June 2002 (rows) and the Envisat classification of June 2005 (columns), presenting numbers of pixels classified as wetland and dryland on both classifications.

		**Radar**		Total
		Wetland	Dryland	

**Optical**	Wetland	1478	2858	4336
	Dryland	1421	2641810	2643231

	Total	2899	2644668	2647567
